# Comparison of memory impairments among two groups of patients with diabetes with different disease durations

**DOI:** 10.1186/1756-0500-5-353

**Published:** 2012-07-16

**Authors:** Mohamad Ali Heidari Gorji, Heshmatollah Ghahremanlu, Mohsen Haghshenas, Mohammad Reza Sadeghi, Ali Morad Heidari Gorji

**Affiliations:** 1 Department of Nursing, Mazandaran Medical Science University, Sari, Iran; 2 Department of Psychology, Mashhad, Iran; 3 Department of Pediatric, Babol Medical Science University, Babol, Iran; 4 Department of Psychology, Mazandaran University of Medical Sciences, Sari, Iran

## Abstract

**Background:**

Modest cognitive impairment has been reported in adults with diabetes. Therefore, we aimed to compare memory impairments among two groups of patients with diabetes with different disease durations.

This study included 120 patients treated at the diabetes clinic at Imam Khomeini Hospital, Ardebil, Iran, over 14 months (2009–2010). The patients were divided into two groups according to their disease duration as >5 years or <1 year (recently diagnosed). The two groups were approximately matched in terms of age and education. Memory impairments were examined using the Wechsler Memory Scale. Data are presented descriptively, and were compared between groups using multivariate analysis of variance.

**Finding:**

Overall, there were no significant differences in total scores or individual subscales between the two groups. However, 59% of all patients had below-average scores on the Wechsler memory questionnaire.

**Conclusion:**

Both groups reported below-average scores on the Wechsler Memory Scale that were independent of disease duration. The present study agreed with the results of other studies showing impaired memory among patients with diabetes. The current findings require further investigation in longitudinal studies.

## Background

Insulin is a key cellular signaling molecule. Patients with diabetes are unable to produce or efficiently utilize insulin, resulting in hyperglycemia [1]. Some studies have explored the relationship between insulin and cognitive disease among patients with diabetes [2,3]. It seems that hyperglycemia may affect cognition and lead to memory defects in daily life in patients with diabetic [4]. Experimental studies have also revealed that insulin can influences memory function in animals [5-7]. The hippocampus is established as the main site of memory formation and learning, and studies have determined the levels of insulin in the hippocampus. Diabetes may result in decreased insulin levels in the hippocampus because of impaired insulin transportation to the hippocampus [8], and may therefore affect memory. The overall changes in glucose levels are related to memory functions [9-11]. Although some studies found no difference between a control and a diabetic group in terms of cognitive function [12], earlier studies have yielded inconsistent results. Furthermore, some chronic diseases, independent of the type of disease, may affect cognitive function. However, in previous studies, the effect disease duration was either overlooked or the findings were contradictory [12,13]. Therefore, in this study, we divided patients with diabetes into two groups according to disease duration to examine the impact of disease duration on memory impairment.

## Methods

### Patients

The present study was a cross-sectional comparative study of patients with diabetes treated over 14 months (2009–2010) at the diabetes clinic of Imam Khomeini Hospital, Ardebil, Iran. This study approved by the research committee at Ardebil Azad Islamic University. The patients were selected by an objective-oriented method based on their disease duration. Based on literature patients with diabetes type 2 considered as a sample. Patients were screened based on the following inclusion criteria: presence of type 2 diabetes, aged 18–60 years, and undergoing usual medical treatment at our diabetes clinic. Patients with co-morbidities, other chronic disease or psychiatric problems were excluded. Overall, 120 patients consented to participate in the study (86 females and 34 males). Of these, 60 patients had diabetes for >5 years and 60 were newly diagnosed, with diabetes duration of <1 year. The two groups were closely matched in terms of age and education.

### Outcomes

Demographic characteristics were evaluated by questionnaire, which covered age, sex, disease duration, education and regular medications. Memory was evaluated by the Wechsler Memory Scale (WMS), a well-established, validated questionnaire. We used the WMS translated by Nasri& Bageri using the WMS, each type of memory is evaluated using two stimuli (auditory and visual) and two types of tasks (recall and recognition). The questionnaire was applied with a 30-minute interval between stages. We also included the Digits test. Eight subtests were estimated Information & Orientation; Spatial Span; Mental Control; Visual Memory; Digit Span; Letter Number; and Word Association. The final memory score was determined as the total of all subscales [10]. Total score of 100 is considered the cutoff value in validity tests for ‘normal’ memory function [14].

### Analysis

SPSS software version 14 (SPSS Inc., Chicago, IL) was used for all analyses. Frequencies and multivariate analysis of variance were used to determine the percentages of below- and above-average scores, and to compare the two groups for each memory questionnaire subscale.

## Findings

We hypothesized that there would be significant differences between the two groups in terms of memory function derived from the WMS subscales. However, as shown in Table 1, there were no significant differences in any subscale or total score between the two groups (*P* > 0.005). Therefore, the null hypothesis was accepted.

**Table 1:** Demonstrate multi variable variance analysis in two different groups on memory subscales

**Sources**	**Variables**	**S**	**Df**	**MS**	**F**	**P**
Groups	Iinformation & Orientation	1/200	1	1/200	088/0	0767/0
	Spatial Span	833/0	1	0833/0	0124/0	0725/0
	Mental Control	008/0	1	008/0	006/0	0938/0
	Visualize memory	208/0	1	208/0	022/0	0844/0
	Word association	1/008	1	1/008	0520/0	0509/0
	Digit Span	133/0	1	0133/0	0225/0	0603/0
	Letter number	8/533	1	8/533	0/096	045/0

There was 45% smoking and no drug abusing in our sample. Among 120 selected subjects 34 cases were male and 86 were female. Mean age was 41/46 _ + 10.16.% and age range were between 30–45. 11% of patients were working and 14.2% were retired and 46.7% of patients was home maker and rest non- working. All participants were literate among them 40.8% had elementary education level and rest higher. Among participants 52% of memory scores lower than normal and 27% were medico rite, only 19% of patient were higher than appropriate.

## Discussion

The present study was conducted to compare the memory function of two groups of patients with diabetes according to disease duration. We found a marked relationship between diabetes and memory, as just over half of the patients (52%) had below-average scores on the WMS. These results were consistent with those reported by Rogers [15] and Sani et al [16], who described that diabetes and altered insulin signaling may affect memory function. In another study [8], glucose fluctuations were shown to disrupt memory formation in rats. However, the results are not consistent with those reported by Amine et al. [17]. However, this difference may be due to the inclusion of adolescents with type 1 diabetes in the study by Amine et al. [17]. Nevertheless, insulin and glucose are necessary to maintain normal brain function; thus, the disruption of insulin action is likely to cause weaknesses in daily memory function. Our current results showed no difference in memory between two groups according to duration of diabetes. However, in a study by Grodstein et al [18], increases in disease duration were found to decrease memory function, although their findings may reflect the age of participants, as that study included older adults. It is already well established that memory is greatly affected by age. Thus, we should interpret our findings in relation to other related studies, particularly those Sani et al [16] and Agostina et al [19], who found that diabetes in any stage of life may negatively affect memory function. In fact, studies have clearly demonstrated that decreases in insulin levels may substantially impair memory function, independent of disease duration [20-22].

## Conclusion

The results of our study and earlier studies [2,4,9,10] have demonstrated a strong relationship between memory problems and diabetes. Thus, patients must be aware and attempt to control their diabetes by following their physician’s advice to prevent diabetes-associated memory impairments.

## Competing interest

The authors declare that they have no competing interests.

## Authors’ contributions

Each author has participated actively and sufficiently in this study. MHG and MRS conceived the idea and design of the study, interpretation of data, and data collection and drafted the manuscript. HG and MH conceived the idea and supervised all work processes and gave advices and revise. MH and AHG made substantial contribution to writing, editing and review of literature. Each author revised critically the manuscript and provided final approval of the version to be published and believes that the manuscript represents honest work.
